# Patient attitude and determinants toward chronic diseases control: A cross-sectional survey in rural China

**DOI:** 10.3389/fpubh.2022.970032

**Published:** 2022-10-13

**Authors:** Yuanyuan Lu, Yuhang Zhao, Xiaofang Shangguan, Benyan Lv, Rui Huang

**Affiliations:** ^1^Maternal and Child Health Hospital of Hubei Province, Tongji Medical College, Huazhong University of Science and Technology, Wuhan, China; ^2^School of Pharmacy, Tongji Medical College, Huazhong University of Science and Technology, Wuhan, China; ^3^Management Institute, Xinxiang Medical University, Xinxiang, China

**Keywords:** chronic disease, cross-sectional survey, attitude, patient satisfaction, rural population

## Abstract

**Objectives:**

The patients' attitude is critical in disease control. This study aims to explore the determinants of patients' attitude and satisfaction.

**Methods:**

A total of 844 patients in the rural areas of Shandong, Henan, and Sichuan provinces with hypertension or diabetes were randomly selected for investigation. The outcome variables were the patients' attitude and satisfaction toward chronic disease control, which were measured through patient self-reported. Binary logistic regression models were used to explore the determinants of patients' attitude and satisfaction.

**Results:**

Teachers were more likely to regard that chronic disease management was helpful in their chronic disease control than that in farmers (OR = 3.994, 95% confidence interval (CI) = 1.309–12.188). Moreover, the probability of health institutions recording chronic diseases changes and guiding rehabilitation for patients regularly is considered helpful 2.688 times more than those that are not. In addition, receiving chronic disease management services can make patients repute that chronic disease management services are helpful in controlling chronic diseases more capably (OR = 1.582, 95% CI = 2.198–10.771). In terms of satisfaction, patients who do not know regular follow-up tend to be dissatisfied with chronic diseases control result (OR = 0.376, 95% CI = 0.192–0.737; OR = 0.592, 95%CI = 0.417–0.841).

**Conclusion:**

The government increases the promotion of chronic disease management in rural areas to improve patients' awareness. Health institutions also can provide diversified services to meet the needs of more people. At last, paying more attention to the timeliness of health services need to be considered to develop a health plan.

## Introduction

Chronic diseases have been becoming the main cause of death and disability in the world (1). Diabetes and cardiovascular disease are major chronic diseases (2). According to the report, the number of diabetes globally has reached 425 million, which will increase to 629 million in 2045 (3). The estimated number of adults suffering from hypertension globally was expected to reach 1.56 billion by 2025 (4). Treatment for characteristics of chronic diseases is long-term and expensive and will consume vast healthcare resources (5). Simultaneously, demand for high-quality healthcare has been greatly and continuously rising with the development of the economy in recent decades. The need for long-term and expensive healthcare, on the one hand, lays a heavy burden on the healthcare system, and on the other hand, greatly threats the equity of the system (6, 7), especially in rural China where the healthcare resources are insufficient (8).

Adhering to chronic disease management is critical to improving health and quality of life for patients (9). It has been suggested for chronic disease control and prevention (1, 10, 11). Focusing on the joint intervention of patients, medical staffs, and medical policies, the chronic care model was proposed in 1998 (12), and was advanced with the expansion and extension of innovative care for chronic conditions. In the United Kingdom, supported by teamwork under chronic disease managers' supervision, ~90% of patients receive healthcare from community health institutions (13). Under the political commitment to universal health coverage in China, central and local governments have funded interventions for hypertension and diabetes. A series of strategies and programs have been developed to control the incremental trend of chronic disease morbidity. These included defining the responsibilities of different health agencies, developing a supportive community environment, training chronic disease staff, providing population-based public health interventions such as establishing health files and providing health promotion services, and individual-based health interventions which include regular follow-up services and guidance in healthy life (14–16). They were included in the national essential public health services (EPHSs) and have been accessible to every patient for free since 2009. The health management services for chronic disease patients mainly include establishing health records, regular follow-up visits, and health promotion (17, 18).

Nevertheless, the effect of chronic diseases on control is dissatisfied by the public (19). The dissatisfaction of patients was that their expectations have not been met and do not achieve their requirements (20). Limited research shows that patients prefer to manage their health by participating in medical decisions. Patient involvement in healthcare has become a national priority. However, the majority of chronic disease interventions are designed without patients in practice (19). Based on knowledge, attitude, belief, practice (KABP), patients' acceptance and satisfaction with medical services are the main factors that affect individual decision-making behavior, and that will affect patients' health (21–23). In addition, the capacity of health systems in rural and urban did not improve at the same rate. In rural areas, EPHS is the main provider of resident health (24). Compared to the contributions of the demand side, most interventions mainly focused on the implementation of the supply side (25). Therefore, this study is conducted to explore the determinants of patient attitudes and satisfaction, including patients' knowledge, attitudes, beliefs, and practices about chronic disease control, to provide information for further improvement in the rural chronic disease management (CDM) system.

## Methods

### Study design

A cross-sectional survey was conducted in rural areas of Henan, Shandong, and Sichuan Province in China, from June to August 2016. Stratified random sampling was used to select rural areas in 53 cities from three provinces. To ensure the quality of the investigation, investigators had been trained before the investigation started. During the implementation of the survey, the quality of the survey was controlled by reviewing the questionnaires on the day. The validity of the questionnaire was evaluated and verified through expert consultation and group discussion. Because the survey questions are simple, and most of them are established facts, the quality of the survey can be guaranteed, and the reliability test has not been carried out.

In total, 997 patients were investigated, and 844 of them provided valid information in the end (the criteria for valid questionnaires were filling time > 5 min and question completion rate ≥ 90.0%). Interviewers were face-to-face conducted with each participant. All participants read consent before they participate in the interview.

### Patient and public involvement

Neither patients nor the public was involved in the design, planning, conduct, or reporting of this study.

### Measurement

The outcome variables were the patients' attitude and satisfaction toward chronic disease control, which were measured through patient self-reported. For the attitude, participants were asked “How about the chronic diseases services provided by health agencies for your disease control?” Possible responses ranged from very helpful, helpful, moderate, and unhelpful to very unhelpful. “Very helpful and helpful” were categorized as helpful, and the rest as unhelpful. Similarly, satisfaction was measured by the answer to the following question: How satisfied you are with the result of chronic disease control? The answers ranged from very satisfied, satisfied, moderate, and dissatisfied to very dissatisfied. Participants were classified as satisfied (containing very satisfied and satisfied) and dissatisfied (containing moderate, dissatisfied, and very dissatisfied). Various demographic, socioeconomic, and chronic disease service variables were included in the analysis based on the literature review. Detailed indicators and codes are shown in Table 1 (26–29).

**Table 1:** Definitions of potential variables.

**Potential variables**	**Definitions**
(1) Gender	1 = male, 2 = female
(2) Occupation	1 = farmer, 2 = worker, 3 = teacher, 4 = government employee, 5 = self-employed or migrant worker, 6 = other
(3) Self-assessment of household income level	1 = high, 2 = middle, 3 = low
(4) Medical insurance	1 = no, 2 = yes
(5) Distance to the nearest health facilities (Km)	1 = “ <1”, 2 = “1–2 (2 not included)”, 3 = “2–3 (3 not included)”, 4 = “3–4 (4 not included)”, 5 = “4–5 (5 not included)”, 6 = “≥5”
(6) Nearest medical institutions	1 = county hospitals, 2 = township hospitals, 3 = village public clinics, 4 = village private clinics
(7) Willingness to seek treatment from village clinics first	1 = yes, 2 = no, 3 = depending on the circumstance
(8) Health institution established health records	1 = yes, 2 = no
(9) Institution provide healthcare information recording	1 = yes, 2 = no
(10) Institutions provide disease changes and guide rehabilitation regularly	1 = yes, 2 = no
(11) Be aware of regular follow-ups	1 = yes, 0 = no
(12) Be aware of health consultation	1 = yes, 0 = no
(13) Be aware of free blood pressure measurement	1 = yes, 0 = no
(14) Received chronic diseases management services	1 = yes, 0 = no
(15) Received health examination	1 = yes, 0 = no
(16) Received free blood pressure measurement	1 = yes, 0 = no
(17) Received regular follow-up	1 = yes, 0 = no
(18) Timeliness of institutional service	1 = timely, 2 = general, 3 = not timely

### Statistical methods

The database was established using EpiData version 3.1 (Atlanta, GA, USA) and double-entered independently to avoid entry errors. All statistical analyses were carried out by SPSS version 24.0 (SPSS Inc., Chicago, IL USA). Chi-square tests were used to explore differences in covariate values between helpful and unhelpful (satisfied and dissatisfied) groups. Associated factors were filtered by the Wald statistic of the backward stepwise method, and finally, statistically significant variables were included in the binary logistic regression model. *P*-values < 0.05 (two-sides) were set as a statistically level in this study.

## Results

### Sample characteristics

The characteristics of patients are shown in Table 2. The average age of participants was 60 years. More than half of them were farmers (60.94%). Approximately 60% of participants were willing to choose the village clinic first, and only 16.67% were reluctant to visit the doctor at the village clinic primary. Most participants (65.65%) knew that primary health institutions provided the measurement of blood pressure and blood glucose freely, while 11.45% of them have ever received chronic disease management. Moreover, 41.9% of participants considered that the services were provided timely.

**Table 2:** The characteristics of participants, the comparison of patient satisfaction regarding chronic disease management, and the self-reported effect of chronic disease control in rural China.

**Characteristics**	**Participants (*N* = 844)**	**Percent (%)**	**Patient's self-reported effect of chronic disease control**			**Patient satisfaction regarding chronic disease management**		
			**Helpful**	**Unhelpful**	***p*-value**	**Satisfied**	**Unsatisfied**	***p*-value**
**Gender**								
Male	376	45.47	174 (44.39)	191 (46.70)	0.511	222 (47.74)	138 (41.94)	0.106
Female	451	54.53	218 (55.61)	218 (53.30)		243 (52.26)	191 (58.06)	
Missing ^*a*^	17		43			50		
**Current job**								
Farmer	504	60.94	235 (59.64)	251 (61.17)	0.009	292 (62.53)	192 (58.01)	0.227
Worker	84	10.16	37 (9.39)	46 (11.19)		40 (8.57)	40 (12.08)	
Teacher	30	3.63	23 (5.84)	5 (1.22)		18 (3.85)	11 (3.32)	
Government employee	12	1.45	8 (2.03)	4 (0.97)		4 (0.86)	7 (2.11)	
Self-employed or migrant worker	54	6.53	26 (6.60)	27 (6.57)		34 (7.28)	18 (5.44)	
Others	147	17.78	65 (16.50)	78 (18.98)		79 (16.92)	63 (19.03)	
Missing	17		39			46		
**Self-assessment of household income level** ^***b***^								
High	115	13.91	70 (17.54)	42 (10.34)	0.006	57 (12.39)	55 (16.52)	0.031
Middle	506	61.19	241 (60.40)	251 (61.82)		299 (65.00)	186 (55.86)	
Low	206	24.91	88 (22.06)	113 (27.83)		104 (22.61)	92 (27.63)	
Missing	17		39			51		
**Medical insurance**								
No	52	6.16	24 (5.94)	23 (5.58)	0.826	35 (7.35)	13 (3.90)	0.041
Yes	792	93.84	380 (94.06)	389 (94.42)		441 (92.65)	320 (96.10)	
Missing	0		28			35		
**Distance to the nearest health facilities (Km)**								
< 1	403	48.73	188 (47.24)	201 (49.63)	0.493	232 (50.00)	157 (47.43)	0.004
1-	160	19.35	76 (19.10)	79 (19.51)		77 (16.59)	74 (22.36)	
2-	156	18.86	82 (20.60)	72 (17.78)		103 (22.20)	48 (14.50)	
3-	54	6.53	25 (6.28)	27 (6.67)		28 (6.03)	23 (6.95)	
4-	25	3.02	16 (4.02)	9 (2.22)		8 (1.72)	17 (5.14)	
>5	29	3.51	11 (2.76)	17 (4.20)		16 (3.45)	12 (3.63)	
Missing	17		41			49		
**Nearest medical institutions**								
County hospital	89	10.79	51 (12.88)	34 (8.35)	0.014	58 (12.53)	27 (8.21)	0.127
Township hospital	204	24.73	106 (26.77)	96 (23.59)		121 (26.13)	76 (23.10)	
Village clinic	388	47.03	184 (46.46)	191 (46.93)		208 (44.92)	164 (49.85)	
Private clinic	144	17.45	55 (13.89)	86 (21.13)		76 (16.41)	62 (18.84)	
Missing	19		41			52		
**First choice of village clinic**								
Willing	497	60.02	250 (62.81)	234 (57.78)	0.317	285 (61.16)	194 (58.79)	0.479
Unwilling	138	16.67	64 (16.08)	70 (17.28)		71 (15.24)	61 (18.48)	
Depending on the circumstances	193	23.31	84 (21.11)	101 (24.94)		110 (23.60)	75 (22.73)	
Missing	16		41			48		
**Health institution establish health records**								
Yes	489	59.42	287 (72.29)	191 (47.04)	0.000	305 (64.89)	169 (51.37)	0.000
No	334	40.58	110 (27.71)	215 (52.96)		165 (35.11)	160 (48.63)	
Missing	21		41			45		
**Health institution record chronic disease changes and guide rehabilitation regularly**								
Yes	258	30.94	192 (47.64)	60 (14.60)	0.000	177 (37.18)	69 (20.78)	0.000
No	576	69.06	211 (52.36)	351 (85.40)		299 (62.82)	263 (79.22)	
Missing	10		30			36		
**Be aware of regular follow-up**								
No	747	90.77	360 (89.55)	373 (91.87)	0.256	416 (88.32)	311 (94.82)	0.002
Yes	76	9.23	42 (10.45)	33 (8.13)		55 (11.68)	17 (5.18)	
Missing	21		36			45		
**Be aware of health education lecture consultation**								
No	620	75.43	281 (69.90)	326 (80.30)	0.001	355 (75.53)	249 (75.91)	0.901
Yes	202	24.57	121 (30.10)	80 (19.70)		115 (24.47)	79 (24.09)	
Missing	22		36			46		
**Be aware of free blood pressure measurement**								
No	285	34.84	113 (28.11)	166 (40.89)	0.000	141 (30.00)	137 (41.77)	0.001
Yes	537	65.65	289 (71.89)	240 (59.11)		329 (70.00)	191 (58.23)	
Missing	26		36			46		
**Receive chronic disease management service**								
No	731	89.04	388 (97.24)	329 (80.44)	0.000	442 (93.05)	273 (81.98)	0.000
Yes	94	11.45	11 (2.76)	80 (19.56)		33 (6.95)	60 (18.02)	
Missing	23		36			36		
**Receive health examination**								
No	575	69.70	276 (69.17)	288 (70.42)	0.700	317 (66.74)	247 (74.17)	0.023
Yes	250	30.30	123 (30.83)	121 (29.58)		158 (33.26)	86 (25.83)	
Missing value	19		36			36		
**Receive free blood pressure measurement**								
No	246	29.82	100 (25.06)	139 (33.99)	0.005	122 (25.68)	119 (35.74)	0.002
Yes	579	70.18	299 (74.94)	270 (66.01)		353 (74.32)	214 (64.26)	
Missing value	19		36			36		
**Receive regular follow-up**								
No	760	92.12	356 (89.22)	387 (94.62)	0.005	436 (91.79)	309 (92.79)	0.601
Yes	65	7.88	43 (10.78)	22 (5.38)		39 (8.21)	24 (7.21)	
Missing	19		36			36		
**Health service timeliness**								
Timely	340	41.87	239 (59.45)	101 (24.69)	0.000	248 (53.68)	81 (24.70)	0.000
General	369	45.44	131 (32.59)	237 (57.95)		178 (38.53)	182 (55.49)	
Not timely	103	12.68	32 (7.96)	71 (17.36)		36 (7.79)	65 (19.81)	
Missing	32		33			54		

a Missing: the missing of the individual variable.

b Self-assessment of household income level: participants are assessed based on where they live.

The comparison of patient satisfaction and self-reported is shown in Table 2. Roughly 58.84% of participants were satisfied with chronic disease management. The significant differences in satisfaction were found in the subgroups of self-reported household income level, whether have medical insurance or not, distance to the nearest health facilities, whether health institutions established health records or not, whether health institutions updated the health record or not, whether health institution record chronic disease changes and guide rehabilitation regularly or not, whether the respondents were aware of the free follow-up and free blood pressure measurement services or not, the timeliness of health service, whether respondents received services of chronic disease management, free blood measurement, and follow-up services or not.

Approximately half of the patients reported chronic disease management was helpful for their health status. The significant differences in the attitude were found among the subgroup of occupation, self-reported household income level, the nearest medical institution, whether health institutions establish health records, whether record chronic disease changes and guide rehabilitation regularly or not, and whether aware of the services of blood measurement and health promotion or not, the timeliness of health services, whether received the services of chronic disease management, free blood measurement, and follow-up services or not.

### Associated factors of patient satisfaction regarding chronic disease management

Results of binary logistic regression analysis are shown in Table 3. It illustrates that satisfaction was significantly associated with patients' awareness of CDM services. Teachers and government employees were more likely to dissatisfy with CDM than farmers (OR = 0.884, 95% CI = 0.352–2.218; OR = 0.195, 95%CI = 0.044–0.867). Patients who did not purchase health insurance and willing to choose a village clinic firstly were unlikely to dissatisfy. Besides that who do not know regular follow-up tends to be dissatisfied (OR = 0.376, 95% CI = 0.192–0.737). Furthermore, patients who do not know free blood pressure measurement services are prone to be dissatisfied (OR = 0.592, 95% CI = 0.417–0.841). The odds of the effect of those who received service timely toward disease control were 4.466 times than who do not (OR = 4.466, 95% CI = 2.603–7.665).

**Table 3:** Factors associated with patient satisfaction regarding chronic disease management.

**Factors**	**Adjusted**			**Unadjusted**		
	**B^*a*^**	**OR^*b*^**	**95% CI^*c*^**	**B**	**OR**	**95% CI**
**Gender (rel** **=** **Female)**						
Male	0.272	1.312	0.949–1.815	0.235	1.264	0.951–1.681
**Current job (ref Farmer)**						
Worker	– 0.627	0.534	0.312–0.914	– 0.419	0.658	0.409–1.057
Teacher	– 0.124^*^	0.884	0.352–2.218	0.073	1.076	0.497–2.328
Government employee	– 1.634^*^	0.195	0.044–0.867	– 0.979	0.376	0.109–1.301
Self-employed or migrant worker	0.148	1.160	0.582–2.309	0.217	1.242	0.682–2.262
Others	– 0.321	0.726	0.466–1.129	– 0.193	0.825	0.565–1.203
**Medical insurance (ref** **=** **Yes)**						
No	0.872^**^	2.392	1.003–5.707	0.670^*^	1.954	1.017–3.752
**Distance to the nearest health facilities (ref** **=** **Over 5 KM) (km)**						
< 1	0.170	1.185	0.485–2.900	0.103	1.108	0.510–2.407
1	0.012	1.012	0.399–2.565	– 0.248	0.780	0.436–1.761
2	0.756	2.129	0.839–5.407	0.476	1.609	0.707–3.666
3	0.097	1.102	0.387–3.138	– 0.091	0.913	0.360–2.314
4	– 0.972	0.379	0.108–1.328	– 1.041	0.353	0.115–1.087
**Nearest medical institutions (ref** **=** **Private clinic)**						
County hospital	0.565	1.760	0.899–3.445	0.561	1.752	0.994–3.088
Township hospital	0.298	1.347	0.808–2.245	0.261	1.299	0.835–2.019
Village clinic	– 0.199	0.820	0.523–1.286	0.034	1.035	0.698–1.533
**First choice of village clinic (ref** **=** **Willing)**						
Unwilling	– 0.646^*^	0.524	0.321–0.855	– 0.233	0.792	0.538–1.168
Depending on the circumstances	– 0.155	0.856	0.571–1.283	– 0.002	0.998	0.707–1.410
**Health institution record chronic disease changes and guide rehabilitation regularly (ref** **=** **No)**						
Yes	0.377	1.458	0.965–2.205	0.814^**^	2.256	1.632–3.119
**Be aware of regular follow-up (ref** **=** **Yes)**						
No	– 0.978^**^	0.376	0.192–0.737	– 0.883^**^	0.413	0.235–0.726
**Be aware of health education lecture consultation (ref** **=** **Yes)**						
No	0.282	1.326	0.895–1.966	– 0.021	0.979	0.705–1.361
**Be aware of free blood pressure measurement (ref** **=** **Yes)**						
No	– 0.523^**^	0.592	0.417–0.841	– 0.515^**^	0.597	0.445–0.803
**Receive regular follow-up (ref** **=** **Yes)**						
No	0.613	1.847	0.953–3.577	– 0.141	0.868	0.512–1.474
**Health service timeliness (ref** **=** **Not timely)**						
Timely	1.497^**^	4.466	2.603–7.665	1.710^**^	5.528	3.427–8.918
General	0.418	1.518	0.932–2.474	0.569^*^	1.766	1.119–2.788

a B, regression coefficient.

* *p* < 0.05,

** *p* < 0.01. The unadjusted models were conducted by univariate regression analysis.

b OR, odds ratio;

c CI, confidence interval.

### Associated factors of the self-reported effect of chronic disease control

Compared to the farmer, the CDM seemed more helpful to the teacher (OR = 3.994, 95% CI = 1.309–12.188). The higher income the patients were, the more helpful to the health status they reported. Besides, the odds of the effect of health records on disease control were 1.539 times greater than those who did not have health records (OR = 1.539, 95% CI = 1.074–2.204). Moreover, updating the health record and guiding patients or rehabilitation regularly was reported more helpful (OR = 2.688, 95% CI = 1.804–4.004). It is noteworthy that those who received chronic disease management services reported more help than that who did not receive them before (OR = 4.866, 95% CI = 2.198–10.771). The effect of those who received service timely to disease control was 2.927 times greater than that of those who did not (OR = 2.927, 95% CI = 1.708–5.015). More details are shown in Table 4.

**Table 4:** Outcome of logistic regression analysis for examining associated factors of self-reported effect of chronic disease control.

**Factors**	**Adjusted**			**Unadjusted**		
	**B^*a*^**	**OR^*b*^**	**95%CI^*c*^**	**B**	**OR**	**95%CI**
**Current job (ref** **=** **Farmer)**						
Worker	– 0.272	0.762	0.431–1.347	– 0.152	0.859	0.538–1.372
Teacher	1.385^*^	3.994	1.309–12.188	1.592^**^	4.913	1.838–13.135
Government employee	0.524	1.688	0.366–7.785	0.759	2.136	0.635–7.188
Self-employed or migrant worker	– 0.394	0.674	0.348–1.305	0.028	1.029	0.583–1.814
Others	– 0.221	0.801	0.517–1.243	– 0.116	0.890	0.612–1.294
**Self-assessment of household income level (ref** **=** **High)**						
Middle	– 0.668^**^	0.513	0.311–0.846	– 0.551^**^	0.576	0.378–0.878
Low	– 0.569^*^	0.566	0.321–0.997	– 0.761^**^	0.467	0.291–0.750
**Health institution establish health records (ref** **=** **No)**						
Yes	0.431^*^	1.539	1.074–2.204	1.077^**^	2.937	2.189–3.940
**Health institution record chronic disease changes and guide rehabilitation regularly (ref** **=** **No)**						
Yes	0.989^**^	2.688	1.804–4.004	1.672^**^	5.323	3.802–7.452
**Be aware of free blood pressure measurement (ref** **=** **Yes)**						
No	– 0.508^*^	0.601	0.391–0.925	– 0.570^**^	0.565	0.421–0.759
**Receive free blood pressure measurement (ref** **=** **Yes)**						
No	0.384	1.469	0.894–2.412	– 0.431^**^	0.650	0.479–0.881
**Receive chronic disease management service (ref** **=** **No)**						
Yes	1.582^**^	4.866	2.198–10.771	2.149 ^**^	8.577	4.490–16.383
**Health service timeliness (ref** **=** **Not timely)**						
Timely	1.074^**^	2.927	1.708–5.015	1.658^**^	5.250	3.256–8.466
General	– 0.026	0.974	0.578–1.642	0.204	1.226	0.767–1.960

a B, regression coefficient.

* *p* < 0.05,

** *p* < 0.01. The unadjusted models were conducted by univariate regression analysis.

b OR, odds ratio;

c CI, confidence interval.

## Discussion

Although the Chinese government has established a relatively wide rural tertiary service network since 2009 (30, 31), findings of this study indicated that < 60% of patients regarded CDM services as helpful and were satisfied with them. Similar findings can be retrieved from a previous study (32). This calls for more attention to the influencing factors of chronic disease management. Among the influencing factors, patient adherence to intervention measures directly affected the prevention and control effect. The average adherence rate of patients with chronic diseases was reported to be ~50% and was lower than that in developing countries (29). Thus, increasing adherence had a greater effect on health than specific medical therapy improvements (26, 29). Another study revealed the underlying reason that patients with chronic diseases tend to have weak awareness of self-management, and at the same time, lack sufficient family support (33). In comparison, this study was more concerned with the impact of variables, such as demographic and socioeconomic, chronic disease management, and timeliness of services on patient attitudes and satisfaction.

The results indicated that the occupation of patients had a significant impact on their attitude and satisfaction. Compared with farmers, teachers tended to consider CDM services helpful to their health. Nevertheless, they were less likely to satisfy with the current services. According to surveys by the National Bureau of Statistics (China), teachers generally receive a higher level of education than farmers do. Previous studies have shown that education is negatively correlated with satisfaction (34). We, therefore, speculated that different attitudes and satisfaction between teachers and farmers may be caused by different levels of education: teachers may have higher expectations and therefore higher standards for good CDM services. However, CDM services in China are currently in the process of transferring from hospital care to primary healthcare (35). In this context, primary healthcare institutions can only provide basic care, such as blood pressure measurement. This is especially the case in rural areas where the shortage of professional health workers is substantial. The significant gap between provision and patient expectation may result in lower satisfaction. Therefore, the professional competence of healthcare professionals, as well as a sufficient number of healthcare professionals and equipment, are critical during the construction of primary healthcare institutions. For integrating chronic disease management services, the “Hospital-Community” model has been adopted to deepen cooperation between the hospital and primary healthcare institutions in many urban areas in south-eastern China (4, 36). However, it is relatively lacking in rural areas. To improve the quality of service for chronic disease management, more attention should be paid to search for more effective and permanent ways for improving the professional ability of healthcare professionals in rural areas in the subsequent research and work plan.

Findings also revealed that patients who were aware of free services were more likely to be satisfied with CDM services than those who did not know. With the rapid development of China, the investment in essential public health services (EPHSs) has also been increasing. The per capita funding standard has increased from 15 RMB in 2009 to 55 RMB in 2018, and the type of services has increased rapidly (37, 38). Patients go to primary healthcare institutions to receive free chronic disease services, which varied substantially to reduce the in-hospital healthcare financial burden (38). These gaps may cause changes in patient behaviors and satisfaction. It is possible for patients to accept these services and be satisfied after they are aware of the content of EPHS. Therefore, while improving the quality of EPHS services, it is also necessary to strengthen publicity so that more people can know the content of service projects and increase the effectiveness of primary healthcare institutions. Although previous traditional media have a positive effect on health promotion, with the coverage of the network, the influence of new media should not be underestimated. It can also be promoted through short videos, in addition to interpersonal networks, such as doctors, family and friends, and village bulletin boards (3).

Finally, the study found that the timely service of primary healthcare institutions had a close relationship with patient attitude and satisfaction. Timely service provision was significantly correlated with a more positive attitude and higher satisfaction than that was not delivered timely. Chronic diseases are usually with longer causes and were cured slowly, and patients have more opportunities to seek health services from healthcare providers (24). It means that timely service could not only solve the patient's problems but also give them psychological comfort. Previous studies reported that the health level of local residents is determined by healthcare institutions (38). However, the medical resources of rural and urban areas vary greatly. According to reports, the health professionals in urban areas were 10.87 per 1,000 population, but only 4.28 in 2017 (39). Therefore, in order to achieve better results, the government needs to place more investment in the construction of primary healthcare institutions.

## Conclusion

Patient adherence is critical in chronic disease management. The attitudes toward chronic disease management and satisfaction with the result of chronic disease control are inextricably linked to their adherence. In this study, we intend to identify factors that influence patient attitudes toward chronic disease management and satisfaction with the result of chronic disease control. The study showed that factors such as awareness, service utilization, timeliness, and initiative of health institutions all have a significant effect on patient attitudes and satisfaction. Based on the survey results, we recommend that the government increase the promotion of chronic disease management services in rural areas (traditional media and new media), provide diversified services to meet the needs of more people, and pay more attention to the timeliness of health services.

This study has several limitations. First, participants only include patients affected by chronic hypertension and/or diabetes, which may neglect patients affected by other chronic diseases. Next, factors such as the self-assessment of household income levels and timeliness of health services are very subjective and difficult to estimate. We have to assume that the evaluation criteria for each participant are the same in the analysis. Finally, patients' attitudes toward chronic disease management and satisfaction with the result of chronic disease control may be affected by the attitudes of the servant, but we have not taken them into account.

## Data availability statement

The datasets presented in this article are not readily available because the data involves personal privacy, please consult the corresponding author for more details. Requests to access the datasets should be directed to RH, hys19810612@163.com.

## Ethics statement

The studies involving human participants were reviewed and approved by Xinxiang Medical College Ethics Committee (ID: XMU71804159). The patients/participants provided their written informed consent to participate in this study.

## Author contributions

BL and RH designed and planned the study. YL and YZ analyzed the data and drafted the article. All authors participated in the survey, completed the data acquisition, participated in data analysis and result interpretation, carefully revised all contents of the manuscript, critically reviewed, and approved the submitted manuscript.

## Conflict of interest

The authors declare that the research was conducted in the absence of any commercial or financial relationships that could be construed as a potential conflict of interest. The reviewer XG declared a shared affiliation with author(s) YL, YZ, XS, and RH to the handling editor at the time of the review.

## Publisher's note

All claims expressed in this article are solely those of the authors and do not necessarily represent those of their affiliated organizations, or those of the publisher, the editors and the reviewers. Any product that may be evaluated in this article, or claim that may be made by its manufacturer, is not guaranteed or endorsed by the publisher.
